# Support for Metastatic Breast Cancer Patients—a Systematic Review

**DOI:** 10.1007/s13187-020-01783-5

**Published:** 2020-06-10

**Authors:** Justyna Bochenek-Cibor, Magdalena Górecka, Dawid Storman, Małgorzata M. Bała

**Affiliations:** 1 Department of Radiation Oncology, St Lukas Hospital, Tarnów, Poland; 2 Students’ Scientific Group of Systematic Reviews, Systematic Reviews Unit, Jagiellonian University Medical College, Kraków, Poland; 3 Chair of Epidemiology and Preventive Medicine, Department of Hygiene and Dietetics, Faculty of Medicine, Jagiellonian University Medical College, Kraków, Poland; 4 Systematic Reviews Unit, Jagiellonian University Medical College, Krakow, Poland

**Keywords:** Breast neoplasms, Needs assessment, Patient satisfaction, Surveys and questionnaires, Systematic review

## Abstract

Our study objective was to evaluate existing evidence on different types of support received by metastatic breast cancer patients as well as the need for support expressed by such patients. We searched Medline and EMBASE up to January 2019 for survey studies that aimed to assess any type of support among women of any age, with metastatic breast cancer diagnosis. Two reviewers independently screened titles and abstracts, then full texts of retrieved records against inclusion/exclusion criteria, and extracted the data and assessed the quality of included studies with AXIS tool. From a total of 2876 abstracts, we selected 100 potentially eligible full-text articles, and finally, we included 12 records reporting on 11 studies. Due to the variability of methods used to measure and define support, it was not possible to quantitatively synthesize data; therefore, we synthesized them narratively. The quality of the included studies was moderate. We found that most patients are satisfied with the received psychosocial, emotional, informational, and medical support. In the analysis of any support received from a certain type of group of people, we found that the majority of patients reported receiving sufficient support from their family, friends, and healthcare providers. Ten studies showed a high need for informational support. If asked about the need for psychosocial, medical, and sexual support, women also declared the need for such support. Our review revealed that the patients generally receive support from their community but they express high need for information and treatment choice. PROSPERO CRD42019127496

**Electronic supplementary material:**

The online version of this article (10.1007/s13187-020-01783-5) contains supplementary material, which is available to authorized users.

## Introduction

Breast cancer (BC) represents a significant public health burden across the globe. Worldwide, 2.1 million newly diagnosed female breast cancer cases were estimated to occur in 2018, accounting for almost 1 in 4 cancer cases among women [1]. About 5–10% of patients in Western countries are initially diagnosed with advanced (ABC) or metastatic breast cancer (MBC) [2]. It is estimated that approximately 20–30% of early BC patients may recur with MBC [3]. The precise number of MBC patients is unknown, as most cancer registries record primary diagnosis only [4, 5].

In 2016, the European School of Oncology established the ABC Global Alliance. It is a platform developed to improve and extend the lives of patients with ABC. In the Global Status of Advanced/Metastatic Breast Cancer 2005–2015 Decade Report [6], the authors stressed the need for holistic, individualized communication about MBC. According to the report, patients declare inadequate communication and understanding. However, so far, no systematic review explored the evidence on support among women with MBC. Therefore, the aim of this systematic review is to evaluate existing evidence on different types of support received by MBC patients as well as the need for support expressed by them.

## Methods

We prepared the protocol for our research before we commenced the study and we registered it on 20 June 2019 in the International prospective register of systematic reviews—PROSPERO (CRD42019127496). In the reporting of the results, we followed the Preferred Reporting Items for Systematic Reviews and Meta-Analyses guidelines.

### Search Strategy

We searched Medline and EMBASE on 18 January 2019 with no language restrictions. Moreover, we searched on portals and websites of professional organizations and reference lists of identified reviews and we contacted experts in the field. We included studies conducted from January 2008 as, after discussion with experts, during the last decade, the progress in the treatment of breast cancer was so pronounced that it might affect results. The search strategy included MESH/Emtree terms and free text words related to metastatic breast cancer, support, and surveys. The search strategy is presented in the Supplementary material.

### Studies’ Eligibility Criteria

The eligibility criteria for our review included the following: (i) target population: women of any age, with metastatic breast cancer diagnosis (TNM stage IV); studies involving patients with different stages of breast cancer or different types of cancer were included if > 50% of participants suffered from advanced breast cancer; (ii) survey studies published from 2008 (last decade), without language restrictions, investigating any type of support received or expressed by participants such as psychosocial, financial, medical, and informational (studies assessing any of them) as defined by the authors of the study; the frequency and intensity of support preferably measured with validated scales; (iii) studies described in more than one paper were all included but analyzed as one study.

We excluded conference abstracts and poster abstracts because they did not provide sufficient data on methods and results.

### Study Selection and Data Extraction

We downloaded search results to reference management software (Mendeley) in order to remove duplicates. Two reviewers (JBC, MG) independently elected articles first on the basis of titles and abstracts using Rayyan application [7] and then based on full text against inclusion/exclusion criteria and resolved disagreements through discussion. Before the screening, reviewers underwent calibration process (of 50 titles and abstracts) twice to check the agreement rate. In both attempts, we reached the pre-assumed 90% agreement rate. Two reviewers extracted data independently and discussed disagreements to reach consensus. Similarly, before extraction, we also carried out the calibration process on 3 papers to rule out major disagreements and we calculated the agreement rate between extractors to be 81%. When we could not reach consensus, we involved a third author (MB).

### Quality Assessment

Two independent reviewers (JBC, MG) assessed the quality of each study using the Appraisal Tool for Cross-Sectional Studies (AXIS) [8]. We chose this tool as it is specifically designed for cross-sectional studies and only includes items relevant to this design. It includes a total of 20 items (see details in Supplementary material). Each study was awarded “yes” if it satisfactorily met a criterion, while “no” was noted if a study did not meet a criterion. As advised in the tool guide, we also recorded “do not know” responses. We resolved disagreements by a discussion between the two reviewers.

### Data Synthesis

We extracted the relevant information from the included studies using a dedicated pre-piloted extraction form, which also included quality assessment. After calibration exercises, we collected the following data: country of the research, study settings, timeframe, study objectives, funding, methodology, and characteristics of the sample population.

The studies defined support in different ways. We extracted the percentage of respondents indicating receiving a certain type of support or expressing the need for such support. Due to substantial variability in the populations including methods used to measure support, different types of support, and different ways of presenting the data, we did not attempt a quantitative synthesis but decided to summarize the data narratively. Because it was not possible to summarize the data quantitatively, we could not explore the potential for publication bias with a funnel plot.

## Results

### Study Characteristic

Our initial searches resulted in 1017 Medline and 2006 EMBASE records. After removing duplicates, we screened 2876 records, which yielded 100 records for full-text assessment. We included 11 studies published in 12 papers (Fig. 1, Supplementary material) [9–20]. Two studies were described in two papers [10, 16, 19, 20] while 1 paper included 2 studies [11]. The list of excluded studies with reasons for exclusion is presented in the Supplementary material. The characteristics of included studies are presented in Table 1.

**Table 1:** Study characteristic

Author year (country)	Study type	Time frame	Tool	Validation	Language of a questionnaire	Funding	Recruitment process	Sampling strategy	Number of MBC patients	Age mean (range)	Race	Education (%)	Marital status	Time since first diagnosis	Number of “yes” in AXIS
Au 2012 (Hong Kong)	Cross-sectional survey	Sep 2008–Oct 2010	SCNS Short-Form, HADS, MSAS SF, CPSQ	Yes	Chinese	NC	Face-to-face	CS	105	253.4 (4–81)	Asian	No formal education or primary–42.9, secondary or above 51.4	Single 14.3, married/cohabiting 64.8, divorced/separated 7.6, widowed 13.3	43.2 ± 53.4 (24.6 months)	14
Brufsky 2017 (USA), Citron 2017 (USA)	Cross-sectional survey	Jun–Aug 2014	MYDC	Pretested	English, Spanish	C	Online, by telephone, by referral	CV	359	Median 53	White 81, Hispanic 8, Black/African-American 5, Asian or Pacific Islander 4, mixed race 1, Native American or Alaskan Native < 1, decline to answer < 1	High school or less 13, Job-specific training program 5, College degree or attended college 59, Graduate degree or attended graduate school 23	Never married 14, married or civil union 58, divorced 13, separated 3, widow/widower 8, living with partner 4	100.6/70 (97.8) mean/median (SD) months	13
Cardoso 2016 (multi^1^)	Cross-sectional survey	Oct 2012–Mar 2013	Count Us, Know Us, Join Us (Count Us) survey	NR	NR	C	Online	NR	1273	NR	NR	NR	NR	NR	8
Cardoso 2016 (multi^2^)	Cross-sectional survey	Nov 2012–Sep 2013	European Here & Now (H&N)	NR	NR	C	Online	NR	158	NR	NR	NR	NR	NR	8
Seah 2014 (USA)	Cross-sectional survey	Oct 2011–May 2012	TINQ-Likert 5-point scale, HADS, Medical Outcomes Study SF-36	yes	English	NC	Face-to-face	CS	52	Median 51.6 (22.4–80.8)	White 92, Black/Haitian/African-American 4, NR 4	High school graduate or GED 12, technical/vocational, some college 10, college graduate 44, post graduate 31	Married/living with domestic partner 66, divorced/separated/widowed/never married 34	NR	12
Dragomir2013 (Romania)	Cross-sectional survey	Jan 2011–Nov 2012	STAI-X1, BDI-IIA and the EORTC QLQ 30 plus BR 23	NR	Romanian	NR	Face-to-face	CV	62	54.37 (30–81)	NR	NR	Married 77.4	NR	12
Reed 2012 (UK)	Cross-sectional survey	NR	FACT-B	Yes	English	NC	Face-to-face, online	CV	235	58 (25–84)	NR	NR	In a relationship 73.2, not in a relationship 24.3, unknown 2.6	< 6 months 26.9%, 6–12 months 19.2%, 1–2 years 26.9%, 2–5 years 22.6%, > 5 years 4.3%, unknown 0.4%	15
Harding 2013 (multi^3^)	Cross-sectional survey	Mar 2011–NR	Being there	NR	NR	C	Online	NR	216	20–> 80	NR	NR	NR	NR	10
Mayer 2010 (multi^4^)	Cross-sectional survey	Sep 2008–Nov 2009	BRIDGE Survey	NR	NR	C	Face-to-face, telephone, mail	CV	1342	Median 55	NR	NR	NR	NR	10
Spence 2015 (Australia)	Cross-sectional survey	Aug-Sep 2014	Authors’ own questionnaire	NR	English	NC	Online	CV	582	< 30–> 80	NR	NR	NR	77% diagnosed with secondary breast cancer within the previous 5 years	12
Espié 2018 (France)	Cross-sectional survey	Sep–Dec 2015	RÉALITÉS	NR	NR	C	On paper	CV	230	Median 60 (32–87)		NR	NR	Min 6 months	9

^1^Argentina, Brazil, Canada, Germany, Hong Kong, India, Lebanon, Mexico, Russia, Taiwan, UK, USA

^2^Austria, Denmark, France, Greece, Italy, Netherlands, Poland, Spain, Sweden

^3^Cyprus, Czech Republic, Denmark, Greece, Hungary, Iceland, Ireland, Lithuania, Poland, Romania, Sweden, UK

^4^Argentina, Australia, Belgium, Brazil, Canada, Egypt, France, Mexico, Poland, Spain, the UK, the USA, and Venezuela

*Abbreviations: BRIDGE, Bridging Gaps, Expanding Outreach Survey; C, commercial; CPSQ, Chinese Patient Satisfaction Questionnaire; CS, consecutive; CV, convenience; HADS, Hospital Anxiety and Depression Scale; MSAS SF, Memorial Symptom Assessment Scale Short-Form; MYDC, Make Your Dialogue Count; NC, non-commercial; NR, not reported; TINQ, Toronto Informational Needs Questionnaire – Breast Cancer*

The included studies enrolled a total of 4614 participants from 32 countries. The sample size varied between 52 and 1342 participants, with a median sample size of 419 participants.

The questionnaires used by the researchers to measure support varied to a great extent.

Results of the quality assessment with AXIS are presented in the Supplementary material. As the tool guide does not specify how to summarize the results, in order to simplify the assessment, we presented the total number of “yes” awarded by each study in the study characteristics. The criteria that were most commonly (in more than half of the studies) not met or not reported included sample size justification, sampling frame, sample selection, response rate, and non-responders categorization. We analyzed both support already received by women and support as an expressed need (Table 2).

**Table 2:** The types of support that were assessed and the proportion of patients reporting receiving or needing these types of support

	Support received by patients		Comment	Support needs reported by patients		Comment
	Number of studies [references]	% reporting receiving sufficient support		Number of studies [references]	% of respondents expressing the need for receiving support	
Any, not further described	7 [11, 13, 16, 18–20]	7–98	See table	0		
Emotional	2	71 [17]–83 [19]	From treating team; one study [17] reported 29% who do not receive enough support	1 [17]	43	The study reported 57% need help with uncertainty about the future
Psychosocial	1 [13]	92	Strong social support	0		
Informational	1 [17]	82	Difference between reported 18% not receiving enough information about treatment	10 [9, 10, 12–16, 18–20]	42 [18]–95 [10]	See table
Medical	1 [17]	76	Satisfactory amount of contact with healthcare professionals	2	32 [17]–38 [9]	More treatment choice
Sexual	0			1 [17]	38–46	Help from the relationship counselor
Financial	0			0		

### Support Received

The authors assessed any type of support received by patients, as well as emotional, psychosocial, informational, and medical support. The highest number of studies (7) reported on any type of support without any further specification of the type of support but with a specified support provider. The majority of patients received support from their family, friends, and healthcare providers, but the minority of them reported satisfaction with support from support groups and social services. The detailed information concerning sources of support is presented in Table 3.

**Table 3:** Reported sources of any support, not further defined

Sources of support	Support assessment (number of studies)	Sufficient support received (% respondents)	Comment
Family	6 [11, 16–19]	57 [17]–98 [19]	
Healthcare providers	4 [11, 14, 18]	73 [18]–85 [11]	One study [14] used Visual Analogue Scale, range 0–10, result 6.0
Friends	3 [11, 18]	59 [11]–87 [18]	Reported 87% [18] concerned support received from family and friends altogether
Support groups	2	33 [17]–36 [11]	
Social services	1	7 [11]	
Anyone	1	77 [18]	Study reported 23% receiving no supportive care

### Support as Expressed Need

The authors assess informational, medical, emotional, and sexual needs for support. Ten out of 11 studies reported on the need for informational support among MBC patients. Most women required information regarding different issues related to their diseases, such as treatment, disease, symptoms, and side effects. The detailed information concerning topics of interest of patients is presented in the Supplementary material. The need for medical support was assessed in 2 studies. The results are consistent: about one-third of MBC patients want to have access to more treatment choices.

## Discussion

Our review revealed that the patients generally receive support from their community but they express high need for information and treatment choice.

This study is the first systematic review in this field, providing a critical summary of the current situation for healthcare providers, policy-makers, and healthcare managers.

In our literature search, we found no data from underdeveloped countries. That stays in accordance with the results of the literature search performed by the authors of the Global Report [6]. They found commonality in many of the personal challenges faced by patients with MBC, but the country in which they were living was the most important factor influencing the appropriate level of support. We also found wide variability in the proportions of patients reporting receiving and needing support. It seems that European patients [11] are less satisfied with the support received from family than globally analyzed patients from North and South America, Africa, and Europe [19]. The results of the assessment of support from healthcare providers stay in accordance irrespectively of origin. Support groups seem ineffective both in Europe and Australia. It is hard to compare the levels of psychosocial support as we only found two studies, one with the smallest number of participants, coming from Romania, and one from Australia. Two authors assessed the emotional support, with the total number of participants being almost 2000 and the results are consistent. It seems that patients from the USA have the highest informational needs [10, 12, 16, 19]. We suppose that is related to higher population awareness and more patients’ involvement in decision-making in the USA than in Europe.

Our research is not free from limitations. The ranges of the tools and measures used in the analyzed research varied to a large extent; therefore, it was not possible to quantitatively synthesize the results of all studies in meta-analysis. Moreover, several studies did not provide data necessary for meta-analysis or data necessary to explore heterogeneity (such as characteristics of the study population). Another concern is a possible selection bias that could be attributed to the subject of the questionnaire, i.e., patients dissatisfied with the support they receive might be especially interested in expressing their opinions.

The lack of validated tools limits the comparability of the results and could even raise concerns about the validity of certain studies. Only three studies reported on the validation of the survey [9, 12, 14] while one form was pretested only [10].

Our quality assessment revealed the moderate quality of the included studies. Most studies did not provide sample size justification, sample selection, nor response rate and non-responder categorization which might have introduced selection bias. Therefore, studies properly planned, using validated tools, explaining sample selections are needed.

Despite our comprehensive search, we cannot exclude the possibility that we missed studies which remained unpublished. Additionally, we excluded studies published only as conference or poster abstracts only, as they did not provide sufficient data on methods and results.

Because quantitative analysis using meta-analysis was not possible, we were unable to assess the publication bias with the funnel plot. Hence, we cannot exclude possible publication bias.

The strength of this study to be underlined is the well-recognized rigorous method of systematic review following the protocol with defined methods registered in PROSPERO.

This systematic review contributes new valuable information to the body of literature in the field of metastatic breast cancer.

## Conclusions

The findings of this review shed light on the needs of MBC patients as well as on the fact that researchers pay little interest in the exploration of the types and amount of support received and expressed by MBC women. The global community must work upon a strong foundation of knowledge on which to take steps forward towards a multi-stakeholder drive for change.

## Electronic Supplementary Material

ESM 1 (DOCX 111 kb)
