# Supplementary material for: Support for Metastatic Breast Cancer Patients—a Systematic Review
Source: J Cancer Educ. 2020 Jun 10;35(6):1061–7. doi: 10.1007/s13187-020-01783-5 (PMC7679297; doi:10.1007/s13187-020-01783-5)
Supplement: Supplementary file 1 — (DOCX 111 kb) [file 13187_2020_1783_MOESM1_ESM.docx]

**Search strategy**

Medline Ovid

#1 'metastatic breast cancer'/exp

#2 metastatic breast cancer.ab,ti,tw,kw.

#3 metastatic breast carcinoma$.tw,sh.

#4 metastatic breast carcinom/exp

#5 metastatic breast neoplasm/exp

#6 metastatic breast tumour/exp

#7 metastas*.ab,ti.

#8 exp Breast Neoplasms/

#9 breast cancer.mp.

#10 8 or 9

#11 1 or 2 or 3 or 4 or 5 or 6 or 7 or exp Neoplasm Metastasis/ or (metasta* or secondar* or spread or advanced).mp.

#12 10 and 11

#13 barrier*.tw

#14 facilitator.tw

#15 obstacle.tw

#16 access*.tw

#17 impediment*.tw

#18 exp health education

#19 health education.tw

#20 exp health behavior

#21 health behavior.tw

#22 exp attitude to health

#23 attitude to health.tw

#24 exp health promotion

#25 health promotion.tw

#26 exp health education

#27 health education.tw

#28 exp health services accessibility

#29 health services accessibility.tw

#30 exp Attitude of Health Personnel

#31 Attitude of Health Personnel.tw

#32 exp Patient Satisfaction

#33 Patient Satisfaction.tw

#34 exp Physician-Patient Relations

#35 Physician-Patient Relations.tw

#36 exp Health Services Needs and Demand

#37 Health Services Needs and Demand.tw

#38 exp Social Support

#39 Social Support.tw

#40 exp Needs Assessment

#41 Needs Assessment.tw

#42 exp Patient Advocacy

#43 Patient Advocacy.tw

#44 physicians practice patterns.tw

#45 exp Delivery of Health Care

#46 Delivery of Health Care.tw

#47 exp access to information

#48 access to information.tw

#49 13 or 14 or 15 or 16 or 17 or 18 or 19 or 20 or 21 or 22 or 23 or 24 or 25 or 26 or 27 or 28 or 29 or 30 or 31 or 32 or 33 or 34 or 35 or 36 or 37 or 38 or 39 or 40 or 41 or 42 or 43 or 44 or 45 or 46 or 47 or 48

#50 12 and 49

#51exp questionnaire/

#52 survey.mp.

#53 questionnaire.mp.

#54 51 or 52 or 53

#55 12 and 49 and 54

EMBASE

#1 'metastatic breast cancer'/exp OR 'metastatic breast cancer'

#2 'metastatic breast carcinoma'

#3 'metastatic breast neoplasm'

#4 'metastatic breast tumour'

#5 'metastatic breast tumor'

#6 metastase

#7 metastas*

#8 #1 OR #2 OR #3 OR #4 OR #5 OR #6 OR #7 or metasta$ OR secondar$ OR spread OR advanced

#9 'breast cancer'/exp OR 'breast cancer'

#10 #8 AND #9

#11 barrier*

#12 facilitator

#13 'obstacles'

#14 access*

#15 impediment*

#16 'health education'

#17 'health behavior'

#18 'attitude to health'

#19 'health promotion'

#20 'health care delivery'

#21 'health personnel attitude'

#22 'patient satisfaction'

#23 'doctor patient relation'

#24 health AND services AND needs AND demand

#25 'social support'

#26 'needs assessment'

#27 'patient advocacy'

#28 physicians AND practice AND patterns

#29 'health care delivery'

#30 'access to information'

#31 'health education'/exp

#32 'health behavior'/exp

#33 'attitude to health'/exp

#34 'health promotion'/exp

#35 'health care delivery'/exp

#36 'health personnel attitude'/exp

#37 'patient satisfaction'/exp

#38 'doctor patient relation'/exp

#39 'social support'/exp

#40 'needs assessment'/exp

#41 'patient advocacy'/exp

#42 'health care delivery'/exp

#43 'access to information'/exp

#44 #11 OR #12 OR #13 OR #14 OR #15 OR #16 OR #17 OR #18 OR #19 OR #20 OR #21 OR #22 OR #23 OR #24 OR #25 OR #26 OR #27 OR #28 OR #29 OR #30 OR #31 OR #32 OR #33 OR #34 OR #35 OR #36 OR #37 OR #38 OR #39 OR #40 OR #41 OR #42 OR #43

#45 #10 AND #44

#46 questionnaire

#47 'questionnaire'/exp

#48 survey*

#49 questionnair*

#50 #46 OR #47 OR #48 OR #49

#51 #45 AND #50

**Fig. 1. PRISMA flowchart**

**(***From:*  Moher D, Liberati A, Tetzlaff J, Altman DG, The PRISMA Group (2009). *P*referred *R*eporting *I*tems for *S*ystematic Reviews and *M*eta-*A*nalyses: The PRISMA Statement. PLoS Med 6(7): e1000097. doi:10.1371/journal.pmed1000097)

Studies included in qualitative synthesis
(n =12 records reporting on 11 studies)

## Included

Full-text articles assessed for eligibility
(n = 100)

Full-text articles excluded (n = 88):

Not population of interest (31)

No intervention of interest (11)

Not a survey study (7)

Conference abstract only (14)

Poster abstract only (25)

Records excluded
(n = 2776)

Records screened
(n =2876)

Records after duplicates removed
(n = 2876)

Additional records identified through other sources
(n = 1)

## Identification

## Eligibility

## Screening

Records identified through Medline and EMBASE searching
(n = 3023)

**List of excluded studies with reasons**

Exluded for population not of interest (31):

1. Applebaum AJ et al.^1^
2. [Børøsund E](https://www.ncbi.nlm.nih.gov/pubmed/?term=B%C3%B8r%C3%B8sund%20E%5BAuthor%5D&cauthor=true&cauthor_uid=25525672) et al. ^2^
3. Wright AA et al.^3^
4. Akechi T et al. ^4^
5. Au A et al.^5^
6. Von Ah D et al.^6^
7. David N et al.^7^
8. Fradelos EC et al. ^8^
9. Edib Z et al.^9^
10. Faller H et al.^10^
11. Rachakonda K et al.^11^
12. Rainbird K et al.^12^
13. Lam W et al.^13^
14. Lam W et al.^14^
15. Li P et al^15^
16. Høyer M et al. ^16^
17. Uchida M et al.^17^
18. Watson M et al^18^
19. Pérez-Fortis A et al^19^
20. Ng R et al^20^
21. Berhili S et al^21^
22. Valdes-Stauber J et al^22^
23. Doubova S et al.^23^
24. Snyder C et al.^24^
25. Ahern T et al.^25^
26. Mehmood T et al.^26^
27. Taha S et al.^27^
28. Wang S et al.^28^
29. Cheung Y et al.^29^
30. Manandhar S et al.^30^
31. Kaal et al.^31^

Exluded for not assessing support (11):

1. StatBite: Global Survey^32^
2. Chiew K et al.^33^
3. Abernethy A et al.^34^
4. Algoe S et al.^35^
5. Attai D et al.^36^
6. Li D et al.^37^
7. Ozzane E et al.^38^
8. Wood R et al.^39^
9. Smith S et al.^40^
10. Stanton A et al.^41^
11. Williamson T et al.^42^

Exluded for not being a survey study (7):

1. Banning M et al.^43^
2. Banning M et al.^44^
3. Danesh M et al.^45^
4. Harmer V^46^
5. Kashian N et al.^47^
6. Uchida M et al.^48^
7. Vilhauer R ^49^

Conference abstract only (14):

1. Hanson A et al.^50^
2. Maejima A et al.^51^
3. Wong A et al.^52^
4. Polite B et al.^53^
5. Lee C et al.^54^
6. Mayer M^55^
7. Hasson-Ohayon I et al.^56^
8. DeHart J et al.^57^
9. Eshraghi L et al.^58^
10. Mileshkin L et al.^59^
11. Caleffi M et al.^60^
12. Sheean P et al.^61^
13. Lewis S et al.^62^
14. Mertz S et al.^63^

Poster abstract only (26):

1. Au A et al.^64^
2. Arnaout A et al.^65^
3. Hanson A et al.^66^
4. Toumeh A et al.^67^
5. Gunawan C et al.^68^
6. Kuznecova G et al.^69^
7. Corneliussen-James D et al.^70^
8. Caplan E et al.^71^
9. Marano E et al.^72^
10. Nael E et al.^73^
11. Caplan E et al.^74^
12. Kuznecova G et al.^75^
13. Ahmed I et al.^76^
14. De Courcy J et al.^77^
15. Wells K et al.^78^
16. Durham K et al.^79^
17. Newton L et al.^80^
18. Caleffi M^81^
19. Marven M et al^82^
20. Mayer et al.^83^
21. Puts M et al.^84^
22. Ko N et al.^85^
23. Canosa R et al.^86^
24. Erdem S et al.^87^
25. Mehmood T^88^

References

1. Applebaum AJ, Stein EM, Lord-Bessen J, Pessin H, Rosenfeld B, Breitbart W. Optimism, social support, and mental health outcomes in patients with advanced cancer. *Psychooncology*. 2014;23(3):299-306. doi:10.1002/pon.3418

2. Børøsund E, Cvancarova M, Moore SM, Ekstedt M, Ruland CM. Comparing Effects in Regular Practice of E-Communication and Web-Based Self-Management Support Among Breast Cancer Patients: Preliminary Results From a Randomized Controlled Trial. *J Med Internet Res*. 2014;16(12):e295. doi:10.2196/jmir.3348

3. Wright AA, Stieglitz H, Kupersztoch YM, et al. United States Acculturation and Cancer Patients’ End-of-Life Care. Deutsch E, ed. *PLoS One*. 2013;8(3):e58663. doi:10.1371/journal.pone.0058663

4. Akechi T, Okuyama T, Endo C, et al. Patient’s perceived need and psychological distress and/or quality of life in ambulatory breast cancer patients in Japan. *Psychooncology*. 2011;20(5):497-505. doi:10.1002/pon.1757

5. Au A, Lam WWT, Kwong A, et al. Validation of the Chinese version of the short-form Supportive Care Needs Survey Questionnaire (SCNS-SF34-C). *Psychooncology*. 2011;20(12):1292-1300. doi:10.1002/pon.1851

6. Von Ah D, Kang D-H. Correlates of mood disturbance in women with breast cancer: patterns over time. *J Adv Nurs*. 2008;61(6):676-689. doi:10.1111/j.1365-2648.2007.04563.x

7. David N, Schlenker P, Prudlo U, Larbig W. Online counseling via e-mail for breast cancer patients on the German internet: preliminary results of a psychoeducational intervention. *Psychosoc Med*. 2011;8:Doc05. doi:10.3205/psm000074

8. Fradelos EC, Latsou D, Mitsi D, et al. Assessment of the relation between religiosity, mental health, and psychological resilience in breast cancer patients. *Contemp Oncol (Poznan, Poland)*. 2018;22(3):172-177. doi:10.5114/wo.2018.78947

9. Edib Z, Kumarasamy V, Binti Abdullah N, Rizal AM, Al-Dubai SAR. Most prevalent unmet supportive care needs and quality of life of breast cancer patients in a tertiary hospital in Malaysia. *Health Qual Life Outcomes*. 2016;14(1):26. doi:10.1186/s12955-016-0428-4

10. Faller H, Brahler E, Harter M, et al. Unmet needs for information and psychosocial support in relation to quality of life and emotional distress: A comparison between gynecological and breast cancer patients. *Patient Educ Couns*. 2017;100(10):1934-1942. http://ovidsp.ovid.com/ovidweb.cgi?T=JS&CSC=Y&NEWS=N&PAGE=fulltext&D=medl&AN=28592366.

11. Rachakonda K, George M, Shafiei M, Oldmeadow C. Unmet Supportive Cancer Care Needs: An Exploratory Quantitative Study in Rural Australia. *World J Oncol*. 2015;6(4):387-393. doi:10.14740/wjon928w

12. Rainbird K, Perkins J, Sanson-Fisher R, Rolfe I, Anseline P. The needs of patients with advanced, incurable cancer. *Br J Cancer*. 2009;101(5):759-764. doi:10.1038/sj.bjc.6605235

13. Lam WWT, Kwong A, Suen D, et al. Factors predicting patient satisfaction in women with advanced breast cancer: a prospective study. *BMC Cancer*. 2018;18(1):162. doi:10.1186/s12885-018-4085-3

14. Lam WWT, Tsang J, Yeo W, et al. The evolution of supportive care needs trajectories in women with advanced breast cancer during the 12 months following diagnosis. *Support Care Cancer*. 2014;22(3):635-644. doi:10.1007/s00520-013-2018-x

15. Li PWC, So WKW, Fong DYT, Lui LYY, Lo JCK, Lau SF. The information needs of breast cancer patients in Hong Kong and their levels of satisfaction with the provision of information. *Cancer Nurs*. 2011;34(1):49-57. doi:10.1097/NCC.0b013e3181ef77a0

16. Høyer M, Johansson B, Nordin K, et al. Health-related quality of life among women with breast cancer – a population-based study. *Acta Oncol (Madr)*. 2011;50(7):1015-1026. doi:10.3109/0284186X.2011.577446

17. Uchida M, Akechi T, Okuyama T, et al. Patients’ Supportive Care Needs and Psychological Distress in Advanced Breast Cancer Patients in Japan. *Jpn J Clin Oncol*. 2011;41(4):530-536. doi:10.1093/jjco/hyq230

18. Watson M, Davolls S, Mohammed K, Shepherd S. The influence of life stage on supportive care and information needs in cancer patients: does older age matter? *Support Care Cancer*. 2015;23(10):2981-2988. doi:10.1007/s00520-015-2665-1

19. Pérez-Fortis A, Fleer J, Sánchez-Sosa JJ, et al. Prevalence and factors associated with supportive care needs among newly diagnosed Mexican breast cancer patients. *Support Care Cancer*. 2017;25(10):3273-3280. doi:10.1007/s00520-017-3741-5

20. Ng R, Verkooijen HM, Ooi LL, Koh W-P. Unmet psychosocial needs among cancer patients undergoing ambulatory care in Singapore. *Support Care Cancer*. 2012;20(5):1049-1056. doi:10.1007/s00520-011-1181-1

21. Berhili S, Kadiri S, Bouziane A, et al. Associated factors with psychological distress in Moroccan breast cancer patients: A cross-sectional study. *The Breast*. 2017;31:26-33. doi:10.1016/j.breast.2016.10.015

22. Valdes-Stauber J, Vietz E, Kilian R. The impact of clinical conditions and social factors on the psychological distress of cancer patients: an explorative study at a consultation and liaison service in a rural general hospital. *BMC Psychiatry*. 2013;13(1):226. doi:10.1186/1471-244X-13-226

23. Doubova S V, Casales-Hernández MG, Perez-Cuevas R. Supportive Care Needs and Association With Quality of Life of Mexican Adults With Solid Cancers. *Cancer Nurs*. 2018;41(2):E1-E12. doi:10.1097/NCC.0000000000000492

24. Snyder CF, Blackford AL, Sussman J, et al. Identifying changes in scores on the EORTC-QLQ-C30 representing a change in patients’ supportive care needs. *Qual Life Res*. 2015;24(5):1207-1216. doi:10.1007/s11136-014-0853-y

25. Ahern T, Gardner A, Courtney M. Exploring patient support by breast care nurses and geographical residence as moderators of the unmet needs and self-efficacy of Australian women with breast cancer: Results from a cross-sectional, nationwide survey. *Eur J Oncol Nurs*. 2016;23:72-80. doi:10.1016/j.ejon.2016.05.001

26. Mehmood T, Smith TJ. Being Critical of Critical Care Given to People With Metastatic Cancer: Get Palliative Care Involved. *J Natl Compr Canc Netw*. 2018;16(9):1157-1158. doi:10.6004/jnccn.2018.7080

27. Taha SA, Matheson K, Paquet L, Verma S, Anisman H. Trust in physician in relation to blame, regret, and depressive symptoms among women with a breast cancer experience. *J Psychosoc Oncol*. 2011;29(4):415-429. doi:10.1080/07347332.2011.582637

28. Wang S, Li Y, Li C, Qiao Y, He S. Distribution and Determinants of Unmet Need for Supportive Care Among Women with Breast Cancer in China. *Med Sci Monit*. 2018;24:1680-1687. doi:10.12659/MSM.905282

29. Cheung YB, Luo N, Ng R, Lee CF. Mapping the Functional Assessment of Cancer Therapy - Breast (FACT-B) to the 5-level EuroQoL group’s 5-dimension questionnaire (EQ-5D-5L) utility index in a Multi-ethnic Asian population. *Health Qual Life Outcomes*. 2014;12(1):180. doi:10.1186/s12955-014-0180-6

30. Manandhar S, Shrestha DS, Taechaboonsermsk P, Siri S, Suparp J. Quality of Life among Breast Cancer Patients Undergoing Treatment in National Cancer Centers in Nepal. *Asian Pacific J Cancer Prev*. 2014;15(22):9753-9757. doi:10.7314/APJCP.2014.15.22.9753

31. Kaal SEJ, Husson O, van Duivenboden S, et al. Empowerment in adolescents and young adults with cancer: Relationship with health-related quality of life. *Cancer*. 2017;123(20):4039-4047. doi:10.1002/cncr.30827

32. StatBite: Global Survey: Women Living With Metastatic Breast Cancer and Clinical Trial Participation. *JNCI J Natl Cancer Inst*. 2009;101(11):779-779. doi:10.1093/jnci/djp149

33. Chiew KS, Shepherd H, Vardy J, Tattersall MHN, Butow PN, Leighl NB. Development and evaluation of a decision aid for patients considering first-line chemotherapy for metastatic breast cancer. *Heal Expect*. 2008;11(1):35-45. doi:10.1111/j.1369-7625.2007.00470.x

34. Abernethy AP, Herndon JE, Coan A, et al. Phase 2 pilot study of Pathfinders: a psychosocial intervention for cancer patients. *Support Care Cancer*. 2010;18(7):893-898. doi:10.1007/s00520-010-0823-z

35. Algoe SB, Stanton AL. Gratitude when it is needed most: social functions of gratitude in women with metastatic breast cancer. *Emotion*. 2012;12(1):163-168. doi:10.1037/a0024024

36. Attai DJ, Cowher MS, Al-Hamadani M, Schoger JM, Staley AC, Landercasper J. Twitter Social Media is an Effective Tool for Breast Cancer Patient Education and Support: Patient-Reported Outcomes by Survey. *J Med Internet Res*. 2015;17(7):e188. doi:10.2196/jmir.4721

37. Li D, McCall LM, Hahn OM, et al. Identification of risk factors for toxicity in patients with hormone receptor-positive advanced breast cancer treated with bevacizumab plus letrozole: a CALGB 40503 (alliance) correlative study. *Breast Cancer Res Treat*. 2018;171(2):325-334. doi:10.1007/s10549-018-4828-5

38. Ozanne EM, Partridge A, Moy B, Ellis KJ, Sepucha KR. Doctor-patient communication about advance directives in metastatic breast cancer. *J Palliat Med*. 2009;12(6):547-553. doi:10.1089/jpm.2008.0254

39. Wood R, Mitra D, de Courcy J, Iyer S. Patient-reported Quality of Life and Treatment Satisfaction in Patients With HR+/HER2- Advanced/Metastatic Breast Cancer. *Clin Ther*. 2017;39(8):1719-1728. doi:10.1016/j.clinthera.2017.07.009

40. Smith SK, Westbrook KE, MacDermott K, LeBlanc MR, Amarasekara S, Pan W. Four Conversations: A randomized control trial of an online, shared decision making curriculum among the metastatic breast cancer community. *J Clin Oncol*. 2017;35(31_suppl):47-47. doi:10.1200/JCO.2017.35.31_suppl.47

41. Stanton AL, Low CA. Dispositional and stressor-related emotion regulation in the context of a chronic, life-limiting stressor. *J Pers*. 2012;80(2):287-311. doi:10.1111/j.1467-6494.2011.00732.x

42. Williamson TJ, Love SM, Clague DeHart JN, et al. Metastatic Breast Cancer Collateral Damage Project (MBCCD): Scale development and preliminary results of the Survey of Health, Impact, Needs, and Experiences (SHINE). *Breast Cancer Res Treat*. 2018;171(1):75-84. doi:10.1007/s10549-018-4823-x

43. Banning M, Tanzeen T. Living with advanced breast cancer: perceptions of Pakistani women on life expectations and fears. *Cancer Nurs*. 2014;37(1):E12-8. doi:10.1097/NCC.0b013e318279e479

44. Banning M, Tanzeem T. Managing the illness experience of women with advanced breast cancer: hopes and fears of cancer-related insecurity. *Eur J Cancer Care (Engl)*. 2013;22(2):253-260. doi:10.1111/ecc.12026

45. Danesh M, Belkora J, Volz S, Rugo HS. Informational needs of patients with metastatic breast cancer: what questions do they ask, and are physicians answering them? *J Cancer Educ*. 2014;29(1):175-180. doi:10.1007/s13187-013-0566-x

46. Harmer V. Advanced breast cancer: “here and now.” *Br J Nurs*. 2013;22(17):990. http://ovidsp.ovid.com/ovidweb.cgi?T=JS&CSC=Y&NEWS=N&PAGE=fulltext&D=med7&AN=24067306.

47. Kashian N, Jacobson S. Factors of Engagement and Patient-Reported Outcomes in a Stage IV Breast Cancer Facebook Group. *Health Commun*. October 2018:1-8. doi:10.1080/10410236.2018.1536962

48. Uchida M, Akechi T. Coping With Advanced Breast Cancer. *Curr Breast Cancer Rep*. 2015;7(3):111-116. doi:10.1007/s12609-015-0188-x

49. Vilhauer RP. Perceived Benefits of Online Support Groups for Women with Metastatic Breast Cancer. *Women Health*. 2009;49(5):381-404. doi:10.1080/03630240903238719

50. Hanson A, Guglielmino J, Ashing K. What Young Women Want: A National Needs Assessment of Young Women Affected by Breast Cancer | Arin Ahlum Hanson | Request PDF. *Psycho-Oncology* . 2013;22:24-25. https://www.researchgate.net/publication/293793369_What_Young_Women_Want_A_National_Needs_Assessment_of_Young_Women_Affected_by_Breast_Cancer. Accessed August 6, 2019.

51. Maejima A, Yoshida S, Kudo S, et al. Preference of end-of-life discussion at diagnosis in patients with advanced/recurrent cancer. *J Clin Oncol*. 2017;35(15_suppl):e21511-e21511. doi:10.1200/JCO.2017.35.15_suppl.e21511

52. Wong A, Hui D, Epner M, et al. Advanced cancer patients’ self-reported perception of timeliness of their referral to outpatient supportive/palliative care and their survival data. *J Clin Oncol*. 2017;35(15_suppl):10121-10121. doi:10.1200/JCO.2017.35.15_suppl.10121

53. Blase N. Polite, Jacob B Allred, Hope S. Rugo, Toni Marie Cipriano, Constance Cirrincione, Sarah J. Gehlert, Electra D. Paskett, Clifford Hudis and EPW, Journal of Clinical Oncolog. Differences in psychosocial factors among diverse women enrolled in a phase III cooperative group metastatic breast cancer trial (CALGB 40502/Alliance). *J Clin Oncol 2013 3115_suppl, 9581-9581* . doi:10.1200/jco.2013.31.15_suppl.9581

54. Lee C, Lieberman M, Faheem F, et al. Evaluation of breast cancer patients’ and oncologists’ expectations for integrative oncology in an ethnically diverse population. *J Clin Oncol*. 2016;34(15_suppl):e12028-e12028. doi:10.1200/JCO.2016.34.15_suppl.e12028

55. Mayer M. IN24 PATIENT PERSPECTIVES ON SYMPTOM CONTROL. *The Breast*. 2013;22:S26-S27. doi:10.1016/S0960-9776(13)70037-7

56. Ilanit Hasson-Ohayon GG. Do You See Light? Hope, Social Support andDistress among Younger and Older WomenDiagnosed with Advanced Breast Cancer. *Psychooncology*. 2010;19(Supl.2)(1-313):155-156.

57. DeHart JNC, Stanton A, Eshraghi L, et al. Metastatic Breast Cancer Collateral Damage (MBCCD) project: Addressing unique needs and identifying patients at highest risk. *J Clin Oncol*. 2017;35(15_suppl):e21592-e21592. doi:10.1200/JCO.2017.35.15_suppl.e21592

58. Eshraghi L, Cooper Ortner H, DeHart JNC, et al. Metastatic Breast Cancer Collateral Damage (MBCCD) project: Think tank of advocates and provider-survivors. *J Clin Oncol*. 2017;35(31_suppl):55-55. doi:10.1200/JCO.2017.35.31_suppl.55

59. Linda R. Mileshkin, Lisa Sheeran, Kate Robins-Browne, Michelle Marven, Karla Gough SA. A national survey of support and information needs of Australian women living with advanced breast cancer. *ournal Clin Oncol*. 2012;30(15_suppl):9124.

60. Caleffi M, Burchardt NA, Scapin HP, et al. Patient-reported outcomes in metastatic breast cancer: QoL, needs, and expectations of survivors. *J Clin Oncol*. 2017;35(5_suppl):228-228. doi:10.1200/JCO.2017.35.5_suppl.228

61. Sheean PM, Kabir C, Rao RD, Hoskins K, Stolley M. Lifestyle habits in females with metastatic breast cancer: A pilot study in support of future interventions. *J Clin Oncol*. 2015;33(15_suppl):e20600-e20600. doi:10.1200/jco.2015.33.15_suppl.e20600

62. Lewis S, Esser M, McCann M, Rowe J, Singh N. OR77 Young and metastatic: addressing the unique needs of advanced breast cancer in young women. *The Breast*. 2012;21:S25. doi:10.1016/S0960-9776(12)70084-X

63. Mertz S, Percassi D. BP27 COUNT US, KNOW US, JOIN US GLOBAL SURVEY: LOOKING AT QUALITY OF LIFE ISSUES AFFECTING ADVANCED BREAST CANCER PATIENTS WORLDWIDE AND THE ROLE OF THEIR CAREGIVERS. *The Breast*. 2013;22:S27-S28. doi:10.1016/S0960-9776(13)70040-7

64. Lam WW, Au AH, Wong JH, et al. Unmet supportive care needs: a cross-cultural comparison between Hong Kong Chinese and German Caucasian women with breast cancer. *Breast Cancer Res Treat*. 2011;130(2):531-541. http://ovidsp.ovid.com/ovidweb.cgi?T=JS&CSC=Y&NEWS=N&PAGE=fulltext&D=med7&AN=21617919.

65. Arnaout A, Kuchuk I, Bouganim N, Verma S, Clemons M. Abstract P5-13-01: Does empowering patients improve accrual to breast cancer trials? In: *Poster Session Abstracts*. Vol 72. American Association for Cancer Research; 2012:P5-13-01-P5-13-01. doi:10.1158/0008-5472.SABCS12-P5-13-01

66. Hanson A, Guglielmino J, Ashing K, Meyers K. PO28 DEFINING THE UNIQUE AND PERSISTENT NEEDS OF YOUNG WOMEN IN THE U.S. LIVING WITH METASTATIC BREAST CANCER THROUGH A MULTI-PHASED NEEDS ASSESSMENT. *The Breast*. 2013;22:S28. doi:10.1016/S0960-9776(13)70041-9

67. Toumeh A, Kundu R, Mohamed I. Abstract P5-15-15: Survival duration and quality of life expectations in patients with metastatic breast cancer: The role of treating oncologists in influencing patients’ expectations of therapy – A questionnaire-based study. In: *Poster Session Abstracts*. American Association for Cancer Research; 2015:P5-15-15-P5-15-15. doi:10.1158/1538-7445.SABCS14-P5-15-15

68. Gunawan C, Vatvani AD, Waren K, Thobias Adiya IDGK, Kurniawan A. 531PAssociation between social support levels with delayed presentations and treatments of symptomatic cancers in Indonesia. *Ann Oncol*. 2017;28(suppl_10). doi:10.1093/annonc/mdx676.030

69. Kuznecova G, Kuznecovs I, Kuznecovs S. 37 Marital Status of Women Renders an Essential Influence On Occurrence of the Relapses of Disease and Life Expectancy of Women with Breast Cancer. *Eur J Cancer*. 2012;48:S51. doi:10.1016/S0959-8049(12)70105-3

70. Corneliussen-James D, Percassi D. Count us, know us, join us global survey: Information needs among women living with metastatic breast cancer. *Eur J Cancer*. 2013;49:S298-S449. doi:10.1016/S0959-8049(13)70062-5

71. Caplan E, Mayer M, Grober S, Hanson A. Silent voices: Information, support and practical services needs for metastatic breast cancer patients. *Psychooncology*. 2010;19(S1):S1-S106. doi:10.1002/pon.1689

72. Marano E, Balena F, I C. Metastatic breast cancer: Life, needs, illness. *Eur J Cancer*. 2014;50(0):175.

73. Nael E, Adamthwaite N. Effectiveness of breast cancer follow up in hospital. *Eur J Surg Oncol*. 2013;39(5):502-503. doi:10.1016/j.ejso.2013.01.185

74. Caplan ES, Mayer M, Grober SE. Silent Voices: accessing and meeting the information, support and practical services needs for women living with metastatic breast cancer. *The Breast*. 2011;20:S52-S53. doi:10.1016/j.breast.2011.08.119

75. Kuznecova G, Kuznecovs S, Kuznecovs I. Targets for psychosocial treatment: Family and work after breast cancer. *Asia Pac J Clin Oncol*. 2012;8:219-358. doi:10.1111/ajco.12030

76. Ahmed I, Harvey A, Amsellem M. Abstract P6-09-09: Perceptions of marginalization in those affected by advanced breast cancer. In: *Poster Session Abstracts*. American Association for Cancer Research; 2012:P6-09-09-P6-09-09. doi:10.1158/0008-5472.SABCS12-P6-09-09

77. de Courcy J, Wood R, Mitra, Iyer S. Satisfaction with cancer treatments in HR + /HER2- metastatic breast cancer patients in a real world setting | OncologyPRO. *Ann Oncol 27 68-99 101093/annonc/mdw365*.

78. Wells K, Thompson J, Marven M. Hope & hurdles: An information resource for women with secondary breast cancer. *Asia-Pacific J Clin Oncol 8232-232*. 2012. https://www.researchgate.net/publication/293812721_HOPE_HURDLES_AN_INFORMATION_RESOURCE_FOR_WOMEN_WITH_SECONDARY_BREAST_CANCER. Accessed August 6, 2019.

79. Durham KS. Po33 Susan G. Komen® Investment in Metastatic Breast Cancer (Mbc). *The Breast*. 2013;22(2013):S30. doi:10.1016/s0960-9776(13)70046-8

80. Newton L, Blackburn M. Improving the standards of care for patients with secondary breast cancer in Bradford, West Yorkshire. *Eur J Surg Oncol*. 2013;39(5):514. doi:10.1016/j.ejso.2013.01.226

81. Caleffi M. Abstract P1-09-18: Perceptions and gaps of women living with advanced breast cancer: Results from the &quot;Count Us, Know Us, Join Us&quot; online survey in Latin America. In: *Poster Session Abstracts*. Vol 75. American Association for Cancer Research; 2015:P1-09-18-P1-09-18. doi:10.1158/1538-7445.SABCS14-P1-09-18

82. Marven M, Sheeran L, Aranda S, Robins-Browne K, Gough K, Mileshkin L. 378 The Support and Information Needs of Women with Advanced Breast Cancer. *Eur J Cancer*. 2012;48:S153. doi:10.1016/S0959-8049(12)70444-6

83. Mayer M, Doan J, Lang K, et al. 5088 POSTER Assessment of Burden of Illness in Women With HER2+ Metastatic Breast Cancer: Findings From a Community Web-based Survey. *Eur J Cancer*. 2011;47:S356-S357. doi:10.1016/S0959-8049(11)71530-1

84. Puts M, Sattar S, McWatters K, et al. What is the role of comorbidity, frailty, and functional status in the decision-making process for older adults with cancer and their family members, oncologists, and family physician? *J Clin Oncol*. 2016;34(3_suppl):92-92. doi:10.1200/jco.2016.34.3_suppl.92

85. Ko N, Festa K, Gunn C, et al. Abstract P3-10-09: Predictors of social support among newly diagnosed breast cancer patients seeking care at an urban safety net academic medical center. In: *Poster Session Abstracts*. Vol 77. American Association for Cancer Research; 2017:P3-10-09-P3-10-09. doi:10.1158/1538-7445.SABCS16-P3-10-09

86. Canosa R, Hornyak K, Percassi D. Count Us, Know Us, Join Us survey: Dialogue needed between healthcare professionals and advanced breast cancer patients in order to make informed treatment decisions. *Eur J Cancer Care (Engl)*. 2014;Volume 50,.

87. Erdem, N.P. Dronov, I.V. Morkovkina, W. Fung, A.F. Frangié, M.H. Hoballah, L.A. Atoui, G. Lin DB. Giving a voice to women with advanced breast cancer: Results from the Count Us global survey from six countries. In: ; 2014.

88. Mehmood T. 516PDeterminants of quality of life in women with metastatic breast cancer. *Ann Oncol*. 2017;28(suppl_10). doi:10.1093/annonc/mdx676.015

**Table 1. Quality assessment**

| **Author** | **Were the aims/objectives of the study clear?** | **Was the study design appropriate for the stated aim(s)?** | **Was the sample size justified?** | **Was the target/reference population clearly defined? (Is it clear who the research was about?)** | **Was the sample frame taken from an appropriate population base so that it closely represented the target/reference population under investigation?** | **Was the selection process likely to select subjects/participants that were representative of the target/reference population under investigation?** | **Were measures undertaken to address and categorise non-responders?** | **Were the risk factor and outcome variables measured appropriate to the aims of the study?** | **Were the risk factor and outcome variables measured correctly using instruments/measurements that had been trialled, piloted or published previously?** | **Is it clear what was used to determined statistical significance and/or precision estimates? (eg, p values, CIs)?** | **Were the methods (including statistical methods) sufficiently described to enable them to be repeated?** | **Were the basic data adequately described?** | **Does the response rate raise concerns about non-response bias?** | **If appropriate, was information about non-responders described?** | **Were the results internally consistent?** | **Were the results for the analyses described in the methods, presented?** | **Were the authors’ discussions and conclusions justified by the results?** | **Were the limitations of the study discussed?** | **Were there any funding sources or conflicts of interest that may affect the authors’ interpretation of the results?** | **Was ethical approval or consent of participants attained?** | **Total number of "yes"** |
| --- | --- | --- | --- | --- | --- | --- | --- | --- | --- | --- | --- | --- | --- | --- | --- | --- | --- | --- | --- | --- | --- |
| Au (2012) | yes | yes | yes | yes | no | no | don'tknow | yes | yes | yes | yes | yes | no | no | yes | yes | yes | yes | no | yes | 14 |
| Brufsky (2017), Citron (2017) | yes | yes | no | yes | no | no | no | yes | yes | yes | yes | yes | no | no | yes | yes | yes | yes | yes | don'tknow | 13 |
| Cardoso (2016) | yes | yes | no | yes | no | no | no | yes | don'tknow | don'tknow | no | no | don'tknow | no | yes | yes | yes | yes | don'tknow | don'tknow | 8 |
|  | yes | yes | no | yes | no | no | no | yes | don'tknow | don'tknow | no | no | don'tknow | no | yes | yes | yes | yes | don'tknow | don'tknow | 8 |
| Seah (2014) | yes | yes | no | yes | no | no | no | yes | yes | yes | yes | yes | no | no | yes | yes | yes | yes | no | don'tknow | 12 |
| Dragomir (2013) | yes | yes | no | yes | no | no | no | yes | yes | no | yes | yes | don'tknow | no | yes | yes | yes | yes | no | yes | 12 |
| Reed (2012) | yes | yes | yes | yes | yes | no | no | yes | yes | yes | yes | yes | no | no | yes | yes | yes | yes | no | yes | 15 |
| Harding (2013) | yes | yes | no | yes | no | no | no | yes | yes | don'tknow | don'tknow | no | don'tknow | no | yes | yes | yes | yes | yes | no | 10 |
| Mayer (2010), Mayer (2010) | yes | yes | no | yes | no | no | no | yes | don'tknow | don'tknow | yes | no | don'tknow | no | yes | yes | yes | yes | yes | no | 10 |
| Spence (2015) | yes | yes | no | yes | no | no | no | yes | no | no | yes | yes | no | yes | yes | yes | yes | yes | yes | don'tknow | 12 |
| Espié (2018) | yes | yes | no | yes | no | no | no | yes | yes | no | no | yes | don'tknow | no | yes | yes | yes | no | no | don'tknow | 9 |
